# Diagnostic value of F-18 FDG PET/CT in fever or inflammation of unknown origin in a large single-center retrospective study

**DOI:** 10.1038/s41598-022-05911-7

**Published:** 2022-02-03

**Authors:** Friedrich Weitzer, Tina Nazerani Hooshmand, Birgit Pernthaler, Erich Sorantin, Reingard Maria Aigner

**Affiliations:** 1 Division of Nuclear Medicine, Department of Radiology, Medical University of Graz, Auenbruggerplatz 9A, 8036 Graz, Austria; 2 Division of Pediatric Radiology, Department of Radiology, Medical University of Graz, Auenbruggerplatz 34, 8036 Graz, Austria

**Keywords:** Diagnosis, Medical imaging, Infectious diseases

## Abstract

Cause determination is challenging in fever or inflammation of unknown origin (FUO/IUO) despite today’s diagnostic modalities. We evaluated the value of F-18 FDG PET/CT in an unselected patient collective. This retrospective nonrandomized single-center study enrolled 300 male and female patients with FUO/IUO. PET/CT findings were compared with final clinical outcomes to determine the sensitivity, specificity, clinical significance, etiological distribution of final diagnoses, impact on treatment, role of white-blood cell count (WBC), and C-reactive protein (CRP). In 54.0% (162/300) PET/CT was the decisive exanimation for establishing the final diagnosis, in 13.3% (40/300) the findings were equivocal and indecisive, in 3.3% (10/300) PET/CT findings were false positive, while in 29.3% (88/300) a normal F-18 FDG pattern was present. Statistical analysis showed a sensitivity of 80.2% and a specificity of 89.8% for the contribution of PET/CT to the final diagnosis. CRP levels and WBC were not associated with PET/CT outcome. PET/CT let to new treatment in 24.0% (72/300), treatment change in 18.0% (54/300), no treatment change in 49.6% (149/300), and in 8.3% (25/300) no data was available. Our study demonstrates the utility of F-18 FDG PET/CT for source finding in FUO/IUO if other diagnostic tools fail.

## Introduction

Fever is one of the most common clinical symptoms among patients across all ages. The initial diagnostic work-up involves patient history, physical examination, extensive laboratory tests including a complete blood-cell count, humoral infection parameters, blood chemistry analysis, liver function tests, urinalysis and chest radiographs. Based on clinical signs and symptoms, imaging studies or other diagnostic approaches such as endoscopy or biopsy of suspected sites of infection will be performed^1,2^.

Numerous recommendations exist on how to diagnose the two most common causes: infections and malignancies. Despite state of the art diagnostic tools the diagnostic work-up in FUO/IUO can be a challenge^2^. Especially invasive or semi-invasive diagnostic methods such as endoscopy and biopsy expose patients to inherent risks, are costly, and time consuming^3^.

Petersdorf and Beeson^4^ were the first to place diagnostic criteria to this syndrome with a prospective study in 1961. Durack DT and Street AC have revised and updated the criteria in 1991^5^. A similar term is inflammation of unknown origin (IUO), which is seen as a sub-class of FUO by some authors.

Over 200 causes for fever are described in the literature; most causes are, besides infection, malignancy, autoimmune inflammatory diseases, while other miscellaneous disorders are less common^3,6^.

Hybrid imaging techniques as 2-deoxy-2[18F]fluoro-d-glucose (F-18 FDG) positron emission tomography (F-18 FDG PET)/ computed tomography (CT) offer many diagnostic advantages by combining metabolic pathophysiological information with anatomic pathological information derived from the CT component. Due to the high sensitivity of detecting early metabolic changes, F-18 FDG PET/CT represents the modality of choice in clinical management especially when other imaging modalities deliver inconclusive results (4).

Despite several single/multicenter studies and meta-analyses focused on the sensitivity of F-18 FDG PET/CT for detecting the etiology in FUO/IUO were published, only a few have included larger patient series over a longer time interval^7–10^.

Study aim was to examine the clinical value of F-18 FDG PET/CT in FUO/IUO by comparing PET/CT with final clinical diagnoses, the impact on patient management and possibly treatment change^1^.

## Patients and methods

The study population retrospectively enrolled male and female adult patients > 18 years without known pregnancy who met at least two of the following criteria for FUO/IUO who underwent F-18 FDG PET/CT in FUO/IUO from January 2012 to August 2017.

Inclusion criteria for FUO based on the criteria used by Robine^11^ and Schönau^12^ were:

- Temperature exceeding 38.3 °C at least three different occasions within three weeks,
- Duration of illness of more than three weeks;
- No cause for fever and illness presented despite significant diagnostic efforts.

IUO was defined by the following characteristics:

- Temperature exceeding 38.3 °C on at least three different occasions within three weeks;
- Duration of illness of more than three weeks;
- Significantly raised inflammatory parameters (including elevated C-reactive protein serum levels (CRP) and White-blood-cell counts (WBC));
- No cause for fever and illness presented despite significant diagnostic effort.

All patients had undergone various radiological imaging modalities and laboratory tests including WBC and CRP levels in the majority of all cases. Patients were included regardless of any comorbidities and/or previous antibiotic and/or previous history of cancer and/or antineoplastic treatment to provide an unselected patient collective.

Patients were divided into six groups according to their etiology:

- Infectious diseases: This group contains patients where infection was either confirmed by PCR and/or microbial blood culture or an infectious cause was determined due to pathognomonic results on PET/CT imaging, differential blood analysis, high CRP levels as well as good response to antibiotic treatment despite negative microbial blood culture.
- Autoimmune/rheumatic diseases: This group contains non-infectious autoimmune diseases and systemic rheumatic diseases, which can occasionally manifest as FUO.
- Malignancy: This group contains solid cancer forms and myeloproliferative diseases.
- Cause unknown: Despite long-term follow-up for at least more than one year and constant episodes of fever, no underlying cause for FUO/IUO presented.
- Spontaneous remission: This group contains patients who met the inclusion criteria however, no clear underlying source presented in PET/CT and other diagnostic methods. Long-term follow-up (at least more than one year) showed spontaneous remission of FUO/IUO.
- Miscellaneous: This group contains various miscellaneous cases.

Although whole-body imaging from vertex to toe is preferable, torso examination from supraorbital crest to mid-thigh was acceptable if limited patient compliance demanded a shortened examination protocol in accordance with European Association of Nuclear Medicine/ Society of Nuclear Medicine and Molecular Imaging (EANM/SNMMI) guidelines^13^. Patients were assorted unsystematically to two different PET/CT systems (Discovery ST, GE Healthcare, Milwaukee, WI, U.S.A.; Biograph mCT, Siemens, Erlangen, Germany).

All patients were fasting a minimum of 6 h and advised against increased physical activity to prevent false positive/negative findings by postprandial or muscular uptake of F-18 FDG. Prior to scanning blood sugar was kept within the accepted range according to the EANM guidelines^13,14^.

For PET scanning 5 MBq/kg body weight F-18 FDG (up to a maximum dose of 440 MBq = 11.9 mCi) was applied intravenously one hour before the image acquisition according to EANM dosage chart^14^. PET images were acquired by discontinuously bed movement (2 min per bed position). Transmission CT scans for attenuation correction were acquired using helical mode with 20 slices without using a contrast agent. Both PET and CT scans were reconstructed with a slice thickness of 3.75 mm. All F-18 FDG PET images were evaluated by two experienced nuclear medicine physicians derived from a pool of five (P.B., G.C., S.T., K.R., and S.S.) in consensual reading. Occasional discrepancies were settled involving the whole team.

PET/CT reports, patient history, hospital discharge summary describing the final cause and laboratory parameters including WBC and CRP (within a range of 14 days) were derived from the hospital information system. Final diagnosis for each patient was established on following diagnostic findings: radiological imaging, laboratory examination, microbial blood culture, and follow-up if available. The clinical course and outcome were considered as gold standard for our study.

Statistical analysis was accomplished with Microsoft Excel Version 2010™ for calculation of sensitivity, specificity, accuracy, positive and negative predictive value, false positive rate and false negative rate. The chi-square test was used to compare composition ration between groups. Differences were considered significant when p < 0.005.

The study protocol including the imaging protocol, the workflow and all other procedures were carried out in accordance with relevant guidelines and regulations, reviewed and approved by the “*Ethikkommision an der Medizinischen Universität Graz*” (= ethics committee at Medical University of Graz). The need for written informed consent was formally waived.

### Ethics declaration

All procedures performed involving human participants were carried out in accordance with relevant guidelines and regulations and the ethical standards of the institutional research committee “*Ethikkommission an der Medizinischen Universität Graz*” (= ethics committee at Medical University of Graz) and with the 1964 Helsinki Declaration and its later amendments or comparable ethical standards. Being a retrospective study the need for written informed consent was formally waived.

## Results

The study population contained 300 patients, 141/300 patients (47.0%) were females and 159/300 (53.0%) were males. Patient age ranged from 18 to 88 years with a median age of 61.08 years (SD = 15.99 years). Whole-body F-18 FDG PET/CT from vertex to toe was performed in 228/300 cases (76.0%) while torso imaging from supraorbital crest to mid-thigh due to limited patient compliance was performed in 72/300 cases (24.0%). 200/300 patients (66.67%) met the FUO characterization and 100/300 patients (33.33%) met the IUO characterization.

**Figure 1:**
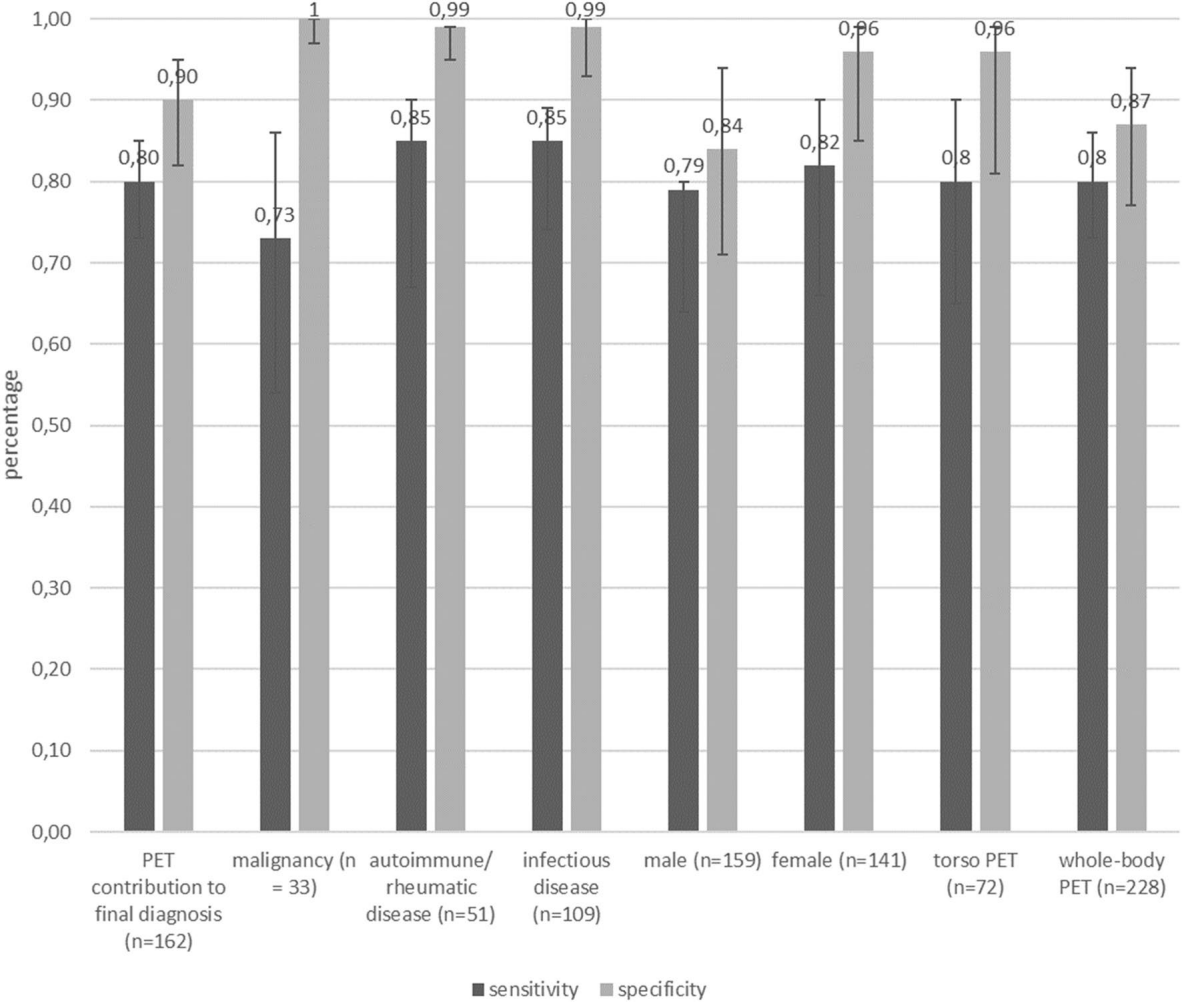
Chart for sensitivity and specificity in study population including 95% Fisher confidence interval. Note the high specificity for malignancy despite a lower sensitivity compared to other causes.

The overall sensitivity and specificity for PET/CT findings essential to establish the final diagnosis were 80.2% and 89.8%, respectively. Sensitivity and specificity between torso PET and whole-body PET and between male and female patients were comparable (Fig. 1).

In 162/300 (54.0%) cases, F-18 FDG PET/CT findings derived from reports matched the final clinical diagnosis (true positive), whereas in 40/300 (13.33%) cases the findings were equivocal and not decisive for determining the final diagnosis (false negative). In 10/300 (3.33%) cases F-18 FDG PET/CT reports could be considered false positive by demonstrating a possible underlying cause for fever which was later outruled by other diagnostic approaches. In 88/300 (29.33%) cases PET/CT showed a normal F-18 FDG pattern (true negative) (Table 1).

**Table 1:** Absolute numbers, percentage and 4 × 4 grid for sorting final diagnoses.

N = 300	Final diagnosis FUO/IUO true	Final diagnosis FUO/IUO false
PET/CT positive	True positive: patients with abnormal PET/CT findings that were essential to establish the final diagnosis (N = 162; 54.00%)	False positive: patients with abnormal PET/CT findings that were outruled by other diagnostic methods (N = 10; 3.33%)
PET/CT negative	True negative: patients with normal PET/CT findings with unknown cause for IUO/FUO (N = 88; 29.33%)	False negative: patients with normal PET/CT findings that received a diagnosis by other diagnostic methods (N = 40; 13.33%)

**Figure 2:**
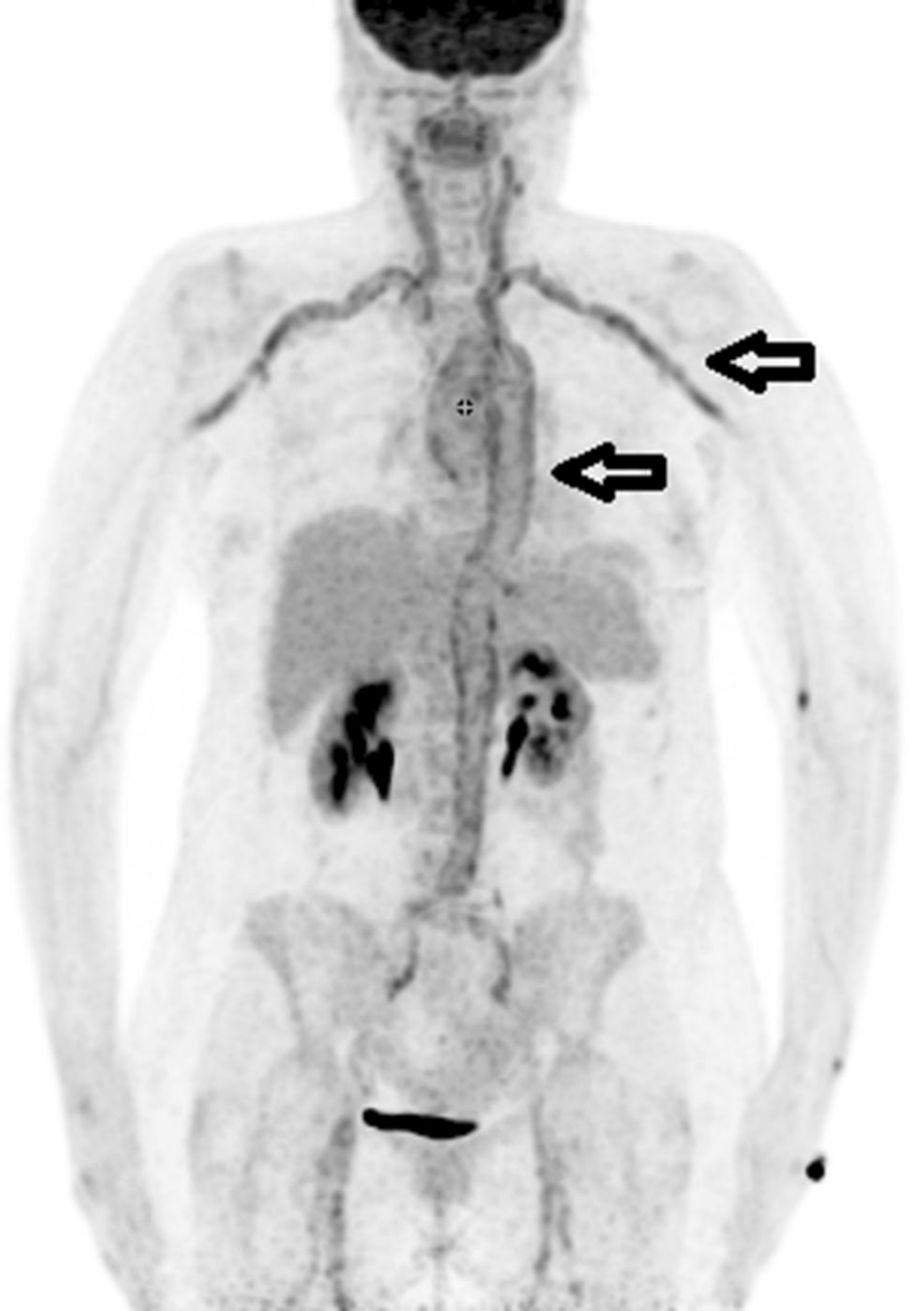
Patient no. 43, 68-year-old female, met the FUO criteria. F-18 FDG PET/CT torso imaging shows longitudinal uptake along the aorta and the large vessels, pathognomonic for giant cell arteritis (arrows). Patient showed only minimal clinical symptoms for vasculitis. Causal anti-inflammatory therapy with oral cortisone was started to which patient responded well.

**Figure 3:**
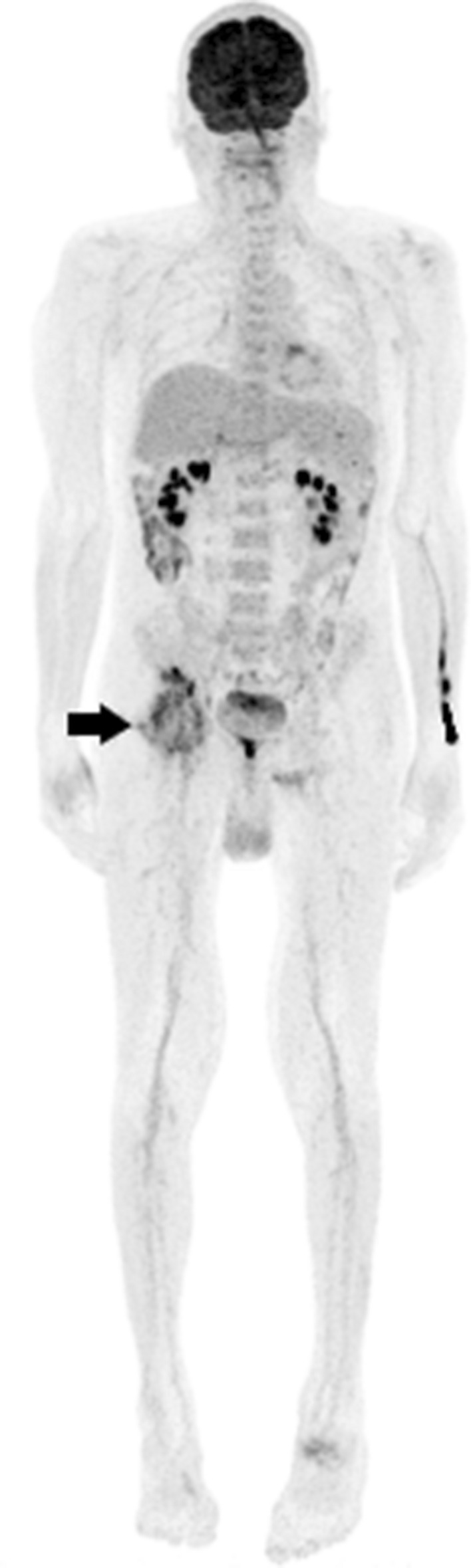
Patient no. 172, 60-year-old male, met the FUO criteria. F-18 FDG PET/CT whole-body imaging shows pathological uptake ad the right hip joint (arrow). Previous clinical examinations suggested activated osteoarthrosis. Biopsy and blood cultures performed after F-18 FDG PET/CT confirmed infective coxitis caused by Staphylococcus aureus. Patient fully recovered after prolonged i.v. antibiotic treatment.

Concerning the distribution of etiology the largest part of final diagnoses were assorted to infectious diseases (109/300 patients, 36.3%) followed by autoimmune/rheumatic diseases (51/300 patients, 17.0%) and malignancy (33/300 patients, 11.0%) (Table 2) Typical examples for infectious and autoimmune/rheumatic diseases are presented in Figs. 2 and 3.

**Table 2:** Distribution and percentage of final clinical diagnoses and sub-groups.

Causes	Patients	Percentage	Final clinical diagnosis
Cause unknown	49	16.3%	Infectious? (15), autoimmune? (15), various diagnoses (12), uncertain (19)
Autoimmune/rheumatic diseases	51	17.0%	Large vessel vasculitis (14), Polymyalgia rheumatica (7), Rheumatoid arthritis (15), various connective tissue diseases (8), various autoimmune disorders (8)
Malignancy	33	11.0%	Solid cancers (21), malignant myeloproliferative disease (12)
Infectious diseases	109	36.3%	Pneumonia (29), prosthetic infection (16), endocarditis (9), soft tissue infection (17), infective arthritis (9), septicemia (14), various infectious diseases (15)
Spontaneous remission	49	16.3%	Exclusion of active infectious disease (28), exclusion of residual malignancy (13), exclusion of various diagnoses (8)
Miscellaneous	9	3.0%	Miscellaneous diseases (9)

PET/CT showed an unexpected high specificity (100%) for diagnosing malignancy despite a lower sensitivity (73%). The large variety of malignancy in our series could further distributed into two sub-groups: solid cancers and myeloproliferative diseases. Overall sensitivity was slightly higher for myeloproliferative diseases than for solid cancer forms (Table 3).

**Table 3:** Laboratory parameters in FUO/IUO.

		PET/CT positive	PET/CT negative	Sensitivity	95% CI (Wald)	95% CI (Fisher)
solid cancer	n = 21	15	6	0.71	(0.52–0.71)	(0.48–0.89)
myeloproliferative disease	n = 12	9	3	0.75	(0.51–0.75)	(0.43–0.95)

*SD* standard deviation, *WBC* range of white-blood-cell, *CRP* C-reactive protein levels.

Patients presented with previous inconclusive or equivocal radiological imaging modalities in 239 cases (79.67%) and none in 61 cases (20.33%) before undergoing F-18 FDG PET/CT. Imaging studies performed before were chest X-rays in 119 cases (39.7%), CT in 124 cases (41.33%), MRI in 61 cases (20.33%) and sonography in 33 cases (11%). In 101 cases (33.67%) patients presented with more than one radiological imaging modality.

Due to F-18 FDG PET/CT results patient management changed to introduction of surgery, chemotherapy, or antibiotic regime in 72 cases (24.0%); significant change in conservative treatment took place in 54 cases (18.0%) while in 149 cases (49.67%) there was no change in the therapeutic regime. In 25 cases (8.33%) the following clinical course was unclear due to outpatient treatment and follow-up, all these patients presented with normal F-18 FDG distribution pattern and were therefore considered true negative. One or more follow-up F-18 FDG PET/CT was performed in 49 cases (16.33%) for monitoring treatment response.

Concerning etiology of infectious diseases, microbiological test methods, mostly blood culture and samples from biopsy revealed a specific pathogen in 46 of 109 cases (42.2%). The remaining cases were clinically diagnosed as infectious diseases due to leukocytosis, high CRP levels and good response to antibiotic treatment.

In 257 (85.67%) patients white-blood cell levels (WBC) and C-reactive protein levels (CRP) were available within 14 days before or after undergoing PET/CT, while in 43 (14.33%) patients neither WBC nor CRP levels were available due to outpatient treatment and follow-up. As demonstrated in Table 4 no statistically significant correlation between WBC and/or CRP levels and PET/CT outcomes could be found.

**Table 4:** Chart for sensitivity and specificity in study population including 95% Fisher confidence interval.

N = 257/300 (85.67%)	White-blood-cell count (*10^11^/L)			C-reactive protein (mg/L)		
	Mean	Range	p	Mean	Range	p
True positive PET/CT	8.59 ± 3.09	2.58–18.38	0.24	60.01 ± 71.44	0.1–313	0.22
False positive PET/CT	10.18 ± 3.46	2.56–17.21	0.24	89.0 ± 66.71	19.1–207	0.22
True negative PET/CT	3.14 ± 4.61	1.78–25.2	0.38	54.69 ± 66.69	0.17–326	0.15
False negative PET/CT	7.90 ± 3.35	2.63–16.68	0.38	48.2 ± 49.37	0.1–163	0.15

Note the high specificity for malignancy despite a lower sensitivity compared to other causes.

## Discussion

The diagnosis of FUO/IUO is a challenge for the responsible physician; therefore, the primary use of the most helpful diagnostic tool is desirable. As mentioned before over 200 diseases can cause FUO/IUO^6^, most of them present with unspecific symptoms or laboratory findings and fever as only symptom. Opposite to conventional imaging, F-18 FDG PET/CT offers high sensitivity in whole-body examinations. Despite the high sensitivity for detecting abnormal glucose metabolism, it is an unspecific tracer molecule, which can be influenced by various diseases including infectious, autoimmune, malignant as well as benign conditions. In the last years several studies and meta-analyses have focused on this rare and challenging topic^9,15^, however the majority of these studies included only a small patient collective.

The presented study is the largest single-center study to our knowledge containing an unselected study population of 300 patients. The study population contained male and female patients (53% vs. 47%) and a wide range of age (18–88 years). The overall sensitivity (80.2%) and specificity (89.8%) was considered adequate, especially the specificity for diagnosing malignancy (100%). The sensitivity and specificity for all groups of final diagnoses was adequate while specificity for malignancy (33 patients) was considerably good (100%). It should be mentioned that despite long-term follow-up in 49/300 cases (16.3%) no cause for FUO/IUO could be found, most likely representing a small but detectable group of long-term FUO. This special condition is marked by fever of unknown origin without any other symptoms over several years and generally has good outcome compared to entire FUO population^16,17^. Our findings are comparable to other data from meta-analysis due to spontaneous remission especially if F-18 FDG-PET/CT is negative^18^.

Long-term outcome was unavailable or unclear in 25/300 cases (8.33%), although these cases presented with a normal FDG pattern.

In our study neither WBC nor CRP levels were associated with PET/CT outcomes. This finding is contradictory to a large retrospective study by Balink et al.^19^ were negative CRP levels of < 5 mg/L were associated with true negative PET/CT findings. Mulders-Manders et al.^20^ reported in a retrospective study including 104 patients no benefit of PET/CT when both inflammatory and body temperature were normal. Another retrospective cohort study including 76 patients showed that F-18 FDG PET/CT is helpful in patients with FUO when CRP, erythrocyte sedimentation rate, ferritin, or leucocytes are elevated^21^. Schönau et al. demonstrated that CRP level over 30 mg/L, age over 50 and absence of fever were significantly associated with diagnostic PET/CT outcome^12^. Predictors and symptoms for diagnostic contribution and successful disease localization by F-18 FDG PET/CT are generally requested. However, in accordance to a recent retrospective study by Tsuzuki et al.^22^ none were identified, although negative PET/CT may be prognostic for spontaneous remission.

The sensitivity (80.2%) and specificity (89.8%) for PET/CT contribution to establish the final diagnosis were adequate when compared to recent retrospective studies and several meta-analyses. Hao et al.^23^ demonstrated comparable results to our findings with a sensitivity of 85% for detecting the focus vs. 81% in our study. Takeuchi et al.^9^ presented in 2016 a nearly identical sensitivity (81% vs. 86%) while the specificity was greater in our study (89% vs. 52%). In contrast, an older meta-analysis including studies from 1998 to 2012 by Besson et al.^15^ demonstrated a significantly lower sensitivity (63% vs 81%) although the usage of PET without CT and different distribution of final diagnoses have to be considered.

While specificity for leucocyte scintigraphy is high in meta-analyses (83% vs 99% in our study if only infectious diseases were concerned), leucocyte scintigraphy in FUO/IUO showed only a sensitivity of 33%, remarkably lower than in our series (85% if only infectious diseases were concerned)^24^. Secondly, the superiority of F-18 FDG PET/CT over Gallium-67 scan was demonstrated by a higher overall sensitivity and a higher specificity (80.2% and 89.8% respectively in our study vs. 60% and 63% respectively in the literature)^23,24^. This is in accordance to a recent study by Kubota et al.^25^ who showed the superiority of F-18-FDG PET/CT over Galllium-67 SPECT. We could not verify the suggestion that F-18 FDG PET/CT is only better in infectious diseases and malignancy, since our data showed good sensitivity (85%) and specificity (99%) in occult autoimmune and/or rheumatic diseases^9^. Nevertheless, we agree to emphasize introducing a more standardized approach for optimal timing, testing and usage of F-18 FDG PET/CT in the diagnostic work-up in FUO/IUO.

Recent studies have shown correlation of F-18 FDG-PET/CT outcomes to the final diagnosis despite different diagnostic approaches prior to F-18 FDG-PET/CT^26^. As early as in 2007/2008 F-18 FDG-PET/CT was recommended for evaluation in FUO/IUO. Larger retrospective studies performed before 2007/2008 introduced F-18 FDG-PET/CT before abdominal and/or chest CT, and invasive techniques such as temporal artery biopsy or bone marrow biopsy in the diagnostic algorithm^27,28^. In these studies F-18 FDG-PET/CT contributed to correct diagnosis in 25%^29^ to 90%^27^ in FUO/IUO with a high negative predictive value (NPV). The recent data asserted this value in correlation with clinical parameters^24,30^. F-18 FDG-PET/CT has proven to be more sensitive in comparison to alternative diagnosis tools such as Gallium-67 scan (with occasional use of SPECT/CT) or 111Indium-labelled leucocytes scintigraphy^31,32^. Newer studies also proved that lack of abnormal F18-FDG uptake can reassure non-existence of conditions thereby avoiding unnecessary additional diagnostic procedures^26^.

Nevertheless the standardization in diagnostic algorithms remains still an obstacle as three older meta-analyses performed between 2007 and 2013 commonly agreed on the consideration of F-18 FDG PET/CT as a second line in FUO work-up^23,33,34^. However, a 2015 pilot study identified F-18 FDG PET/CT as a cost-effective imaging routine in reducing the costs of further procedure and duration of hospitalization (€12,614 compared to €5,298 per FUO patient in the Netherlands)^35^. These findings were backed up by prospective observational study by Cachot et al.^36^ in 2021. Our study confirms these findings despite an unselected study population.

Common limitations of these retrospective studies are limited numbers of patients with heterogeneous diseases^9,26^. Several systematic reviews and meta-analyses have attempted to improve this, including data from the prospective studies. Another critical point is the heterogeneity of population and diseases worldwide making comparison especially with Chinese retrospective studies difficult due to a higher percentage of cases with tuberculosis in up to 17% in IUO and FUO^37^, which is not the case in a Western and especially Middle-European study population.

This study has two limitations: First, being a pure retrospective study there is an inevitably chance of selection bias for included cases. Additionally other diagnostic means and pathways were needed to confirm abnormal PET/CT findings therefore adding further bias. The choice of an unselected study population may be considered as a possible limitation; however, to our view a non-selected study population is the best representation of the daily diagnostic challenge a clinician is most likely to face.

Second, torso imaging from supraorbital crest to mid-thigh was performed in 72/300 cases (24.0%). Based on this abbreviated protocol in limited patient compliance there may be a drawback by not examining suspected areas especially in patients with autoimmune diseases like arthritis and large-vessel vasculitis. However, similar sensitivity in both torso and whole-body examinations (80% vs. 80%) was demonstrated, while specificity was unexpectedly higher in torso examinations (96% vs. 87%). Severely ill patients or higher disease activity therefore limiting patient’s compliance may cause this finding.

## Conclusions

F-18 FDG PET/CT is a helpful method in the diagnosis of FUO. Our study clearly showed an overall high sensitivity and specificity compared to several meta-analyses. Therefore, the early use of F-18 FDG PET/CT should be considered if other diagnostic means fail. Neither elevated CRP nor WBC levels are associated directly with the true positive results. Nevertheless, the optimum timing for the performing as well as the standardization of F-18 FDG PET/CT as a first-line work-up in FUO/IUO patients is still debatable (Supplementary Information S1).

## Supplementary Information

Supplementary Information.
